# Negativity Bias in Dangerous Drivers

**DOI:** 10.1371/journal.pone.0147083

**Published:** 2016-01-14

**Authors:** Jing Chai, Weina Qu, Xianghong Sun, Kan Zhang, Yan Ge

**Affiliations:** 1 Key Laboratory of Behavioral Science, Institute of Psychology, CAS, Beijing, China; 2 University of Chinese Academy of Sciences, Beijing, China; University of Pennsylvania, UNITED STATES

## Abstract

The behavioral and cognitive characteristics of dangerous drivers differ significantly from those of safe drivers. However, differences in emotional information processing have seldom been investigated. Previous studies have revealed that drivers with higher anger/anxiety trait scores are more likely to be involved in crashes and that individuals with higher anger traits exhibit stronger negativity biases when processing emotions compared with control groups. However, researchers have not explored the relationship between emotional information processing and driving behavior. In this study, we examined the emotional information processing differences between dangerous drivers and safe drivers. Thirty-eight non-professional drivers were divided into two groups according to the penalty points that they had accrued for traffic violations: 15 drivers with 6 or more points were included in the dangerous driver group, and 23 drivers with 3 or fewer points were included in the safe driver group. The emotional Stroop task was used to measure negativity biases, and both behavioral and electroencephalograph data were recorded. The behavioral results revealed stronger negativity biases in the dangerous drivers than in the safe drivers. The bias score was correlated with self-reported dangerous driving behavior. Drivers with strong negativity biases reported having been involved in mores crashes compared with the less-biased drivers. The event-related potentials (ERPs) revealed that the dangerous drivers exhibited reduced P3 components when responding to negative stimuli, suggesting decreased inhibitory control of information that is task-irrelevant but emotionally salient. The influence of negativity bias provides one possible explanation of the effects of individual differences on dangerous driving behavior and traffic crashes.

## Introduction

In the driving literature, dangerous drivers share three driving behavior characteristic: intentional physical or psychological aggression, risk taking while driving and negative emotions (e.g., anger and anxiety) during driving [1, 2]. These behaviors are dangerous because they are significantly correlated with involvement in crashes and crash-related conditions, such as loss of concentration, near misses, loss of vehicle control and the imposition of fines or points [3–6]. Previous studies have found significant differences between dangerous drivers and safe drivers in terms of their personalities and traits [7, 8], cognitive abilities [9] and information processing [10–12]. For example, drivers with crash histories perform worse on tests of both visual and auditory selective attention in terms of switching errors and omission errors compared with drivers without crash histories [13]. In recent years, researchers have increasingly focused on the relationships between negative emotions and dangerous driving behaviors [1, 14–17].

The literature in this field primarily focuses on the effects of emotion-related personality traits on driving behaviors. For example, driving anger is thought to critically contribute to increases in traffic collisions and risky behaviors, such as speeding and driving aggressively [17–23]. Studies suggest that anger may interfere with attention, resulting in more superficial assessments of driving circumstances and the subsequent underestimation of potential hazards [24, 25]. Anxiety and fear, which one-fifth of drivers experience [26, 27], have been found to be related to a wide variety of dangerous driving behaviors [15] that decrease driving safety and efficiency by increasing the cognitive workload, consuming cognitive resources [28] and reducing the amount of attention available for ongoing tasks [29]. In addition, studies have shown significant relationships between emotion-related personality traits and dangerous driving behaviors. Drivers with higher anxiety-related personality trait scores report more crashes [15, 30, 31]. Additionally, trait anger is positively associated with dangerous driving behavior and crashes [17]. Furthermore, researchers have found that individuals with higher levels of trait anger may evaluate anger-inducing inputs more negatively and respond more intensely to threatening stimuli compared with individuals with lower levels of trait anger [32, 33]. In a simulator study, drivers with high and low levels of anger traits differed in their evaluations of high- and low-anger-provoking situations. Anger-prone drivers reported feeling more anger and frustration in less-anger-provoking situations and were more likely to speed and overtake other drivers compared with non-anger-prone drivers [34]. These results imply significant individual differences in emotional information processing between drivers with differing personality traits; however, no research has directly explored the differences in emotional processing between dangerous and safe drivers.

Negativity bias is a phenomenon in which negative events are more salient and demand attention more powerfully than neutral or positive events, and it is an important index in the study of emotional processing [35, 36]. Individuals with a stronger negativity bias tend to exhibit more negative attitudes toward a given negative description and to respond more intensely to negative input than do individuals with weaker negativity biases [37]. Individual differences in negativity bias were initially used as an index in psychopathology, primarily in patients with emotional disorders [38–40]. Studies have observed significant emotional bias effects in individuals with clinical disorders, and these effects appear to be unstable or even absent in healthy people. Individuals with higher levels of trait anxiety are disturbed more easily when processing emotional stimuli compared with less anxious individuals [41]. As mentioned above, dangerous drivers were found to have higher anger and anxiety traits [15, 30, 31, 34], but no research has explored whether dangerous drivers process emotional information differently than safe drivers do. Exploring the relationship between dangerous driving and emotional information processing could help us understand the cognitive processing of dangerous drivers.

In addition, previous studies have shown that subconscious information processing significantly influences our attitudes and behavior [42–44]. The emotional Stroop task is an implicit paradigm that is widely used to explore attentional biases during the processing of emotional stimuli [32, 45, 46]. In this paradigm, participants are not required to categorize emotional stimuli based on valence; instead, they are asked to name the color of the frame around a picture stimulus. Compared with explicit paradigms, which typically ask participants to evaluate the valence of pictures, such implicit tasks may elicit emotional responses that more closely resemble natural situations [47] because negative events often occur unpredictably and are processed unconsciously in real driving environments. Therefore, we used the emotional Stroop task to explore how dangerous drivers process emotional information.

Additionally, event-related potentials (ERPs) can be used to explore the neural mechanisms of information processing. Based on previous studies, the negativity bias effect is not observed in behavioral measurements; but the results using the ERP technique have revealed significant biases during the processing of negative stimuli, ranging from early to late ERP components [46–52]. Thus, ERPs provide an effective means of identifying subtle changes that occur during emotional processing tasks. These findings indicate that the influence of negative emotion might extend across the entire processing period. P3, a major positive wave usually occurred around 300ms after stimulus, is a component that is commonly considered to reflect the implicit processing of affective stimuli [50, 53–55]. However, the reported effects of emotional information processing on the corresponding ERP components are inconsistent. Some studies have found that while participants respond to the emotionally irrelevant features of stimuli, the P3 amplitudes elicited by negative stimuli are reduced relative to those elicited by neutral or positive stimuli [47, 52, 56]; in contrast, other studies have reported no differences in the P3s elicited by stimuli with different emotional valences [46, 57]. P3 amplitudes might be indicative of an inhibitory process that is involved in controlling task-irrelevant but emotionally salient features [47, 53, 58]. Compared with the early components, such as N1 or P1, P3 is indicative of more controlled and endogenous information processing stages [59–61] and may be related to cognitive resource allocation during driving [62–64].

In summary, previous studies have suggested that drivers with higher levels of trait anger exhibit more dangerous driving behaviors and that individuals with higher levels of trait anger are more likely to show greater negativity bias than those with lower levels of trait anger. However, no previous research has directly explored the relationship between dangerous drivers and their negativity biases. We hypothesized that compared with safer drivers, dangerous drivers may be more sensitive to negative input (as indicated by stronger negativity biases). Therefore, the purpose of this study was to examine the difference in negativity bias between dangerous drivers and safe drivers. We used penalty points as the criterion to distinguish dangerous drivers from safe drivers because previous studies has proved the significant correlation between points and crash involvement [65–66]. We hypothesized that dangerous drivers and safe drivers would exhibit distinct negativity biases in the emotional Stroop task, with the behavioral and ERP results expected to show stronger negativity biases in dangerous drivers than in the safe drivers. Furthermore, we anticipated that negativity bias would be related to dangerous driving behaviors and crash involvement.

## Methods

### 2.1 Participants

Forty-two non-professional drivers were recruited from an open-access online job website (http://bj.58.com/) and from the campus of the Institute of Psychology of the Chinese Academic of Sciences. All of the drivers had had their driver’s license for at least 3 years, and their driving experience exceeded 30,000 kilometers. The participants were divided into two groups according to the penalty points that they had accrued within the previous year. Eighteen drivers who had been penalized 6 or more points within the last year were assigned to the dangerous driver group, and 24 drivers who had accrued 3 or fewer penalty points were assigned to the safe driver group. Penalty points are recorded by the Beijing Traffic Management Bureau and can be tracked online by the driver’s license. For example, driving without wearing a seatbelt results in a 3-point penalty, and running a red light results in a 6-point penalty. If a person receives 12 penalty points within one year, then his/her driver’s license is suspended. All the participants signed their informed consent before the formal experiment. They completed this experiment voluntarily and anonymously. All their information was strictly confidential and can only be used for scientific research. They received some cash for participating. The Ethical approval was given by the Institutional Review Board of the Institute of Psychology, Chinese Academy of Sciences.

### 2.2 Measurement of driving behavior

Dula Dangerous Driving Index (DDDI). This scale is used to assess an individual’s propensity for dangerous driving [1]. We used the Chinese version of the DDDI, which was translated and proven to have sufficient reliability and validity [4]. This scale exhibited excellent internal consistency, with a Cronbach’s alpha coefficient of 0.898 for all 28 items. The DDDI includes four subscales: Risky Driving (RD, 10 items, α = 0.782), Negative Cognitive/Emotional Driving (NCED, 9 items, α = 0.802), Aggressive Driving (AD, 7 items, α = 0.775) and Drunk Driving (DD, 2 items, α = 0.634). Drivers evaluate features of their everyday driving on a 5-point Likert scale that ranges from 1 (“never”) to 5 (“always”). The DDDI score was also used as an evaluation criterion for classifying the participants as dangerous or safe drivers.

Additionally, information on the participants’ driving experience and traffic violations was collected using self-report questionnaires with items related to the number of years since acquiring a driver’s license, the total driving mileage, the weekly driving mileage, the penalty points and fines acquired within the last year, and the number of crashes during the last three years.

### 2.3 Materials in the emotional Stroop task

All of the pictures used in the emotional Stroop task were adopted from the International Affective Picture System (IAPS) [67]. The task used 20 neutral pictures and 20 negative pictures, which were classified according to their valence and arousal level. For example, a neutral picture may present a common chair or desk, and a negative picture may depict an attacking snake or an injured child. The valences of the negative pictures were significantly lower than neutral pictures (mean for negative pictures = 2.66; neutral mean = 4.92, *t* = 13.66, *p*<0.001), and the arousal levels of the negative pictures were significantly higher than neutral pictures (mean for negative pictures = 1.60; neutral mean = 1.28, *t* = 3.713, *p*<0.001) according to the IAPS instruction manual and affective ratings, in which each picture was measured by a 9-points Likert scale for every dimension [67]. Each picture, whether negative or neutral, had either a red or a blue border. Each picture was presented twice, once with a red border and once with a blue border; thus, the entire formal experiment included 80 trials. The pictures were 53 mm by 40 mm in size (degree of visual angle = 6.10° by 4.58°), and they were shown on a 17-inch flat cathode ray tube (CRT) display with a resolution of 1280*768, a luminosity of 80, a contrast ratio of 60 and a refresh rate of 60.

### 2.4 Procedures

When the participants arrived at the laboratory, they signed the informed consent form and completed the DDDI and a general questionnaire regarding sociodemographic information and driving experience. They then completed the emotional Stroop task. Six trials with neutral pictures were used as practice before the formal experiment began. The formal experiment consisted of 80 trials; half of the trials were affectively neutral, and the other half were affectively negative. In each trial, a white cross was presented in the middle of the screen as a fixation point for a random duration of 600 to 1000 ms. An emotional picture with a red or blue border was then presented in the middle of the screen until the participant chose a response. The participants were instructed to respond based on the color of the picture’s border and to ignore the picture’s content. They were instructed to press the “f” key on the keyboard using their left index finger when they saw a red border and to press the “j” key with their right index finger when they saw a blue border. The left/right-blue/red pairings were counterbalanced among the participants. When the participant responded correctly, the program presented a blank screen for 800 ms and then automatically began the next trial. When the participant responded incorrectly, feedback in the form of the word “incorrect” in red print was presented for 800 ms before the next trial began. The background of the experiment was black. The entire emotional Stroop task required approximately 10 minutes to complete.

### 2.5 EEG recording and analysis

The EEG data were collected during the entire emotional Stroop task, which were recorded from 64 scalp sites using tin electrodes mounted in an elastic cap arranged according to the 10–20 international placement system (Neuroscan Labs, Sterling, Virginia, USA). The online reference was the left mastoid. A horizontal electrooculogram (EOG) was recorded from the left to the right orbital rim, and the vertical EOG was recorded from the supra-orbital to the infra-orbital positions of the left eye. The electrode sites on each participant's face and mastoid were gently cleansed with cotton balls and alcohol. The impedances of the EEG electrodes were below 10 kΩ. The EEG data were amplified with a bandpass filter of 0.05–100 Hz and digitized at 1000 Hz.

The EEG data were processed using Neuroscan 4.5 software and re-referenced to the linked mastoid offline. Ocular artifacts were removed using a regression procedure implemented with the Neuroscan software [68]. The filter was set to low pass (30 Hz, 24 dB/oct) in the zero phase shift mode. Each picture was a stimulus in this study. The EEG data were segmented into 900 ms periods beginning 100 ms prior to the stimulus presentation and ending 800 ms after the target stimulus onset. The first 100 ms was used as a reference for baseline correction. Epochs exceeding ±75 μV were considered artifacts and were rejected. The average responses were obtained for each condition for further analyses. Behaviorally incorrect trials were excluded from the analysis. Trials with reaction times exceeding 1500 ms or falling below 150 ms were also excluded.

The ERP components were identified and analyzed based on a visual inspection of the grand average ERPs. The stimulus-locked P3 (the second major positive wave) had a mean amplitude between 350 and 500 ms post-stimulus onset relative to the baseline. According to previous work, the P3 waveforms during the emotional Stroop test are distributed in the centro-parietal to parietal area. Therefore, the following 9 electrode sites were selected for the statistical analyses: C3, Cz, C4, CP3, CPz, CP4, P3, Pz, and P4. We performed separate 2×2 mixed-design ANOVAs that included the following factors: driver group (dangerous vs. safe; between-subject design), emotional picture (neutral vs. negative; repeated measure). The degrees of freedom of the *p* values were adjusted according to the Greenhouse-Geisser correction. The Bonferroni correction was used for multiple comparisons.

## Results

### 3.1 Participants

One male driver assigned to the dangerous driver group was excluded from the final analysis because his DDDI score was lower than the scores of all drivers in the safe driver group, suggesting that he may not have answered the questionnaire honestly. The mean reaction times of three participants (including 2 dangerous drivers and 1 safe driver) exceeded three standard deviations above the average reaction time, and these participants were also excluded from further analyses. A total of 15 participants from the dangerous driver group (7 males and 8 females) and 23 participants from the safe driver group (12 males and 11 females) were analyzed. Eighty percent of the dangerous drivers were penalized for speeding, and 53.33% of them were penalized for running a red light.

No significant differences in the demographic factors or driving experience of the dangerous and safe drivers were observed. However, there were significant differences between the two groups in the numbers of crashes within the past 3 years. The dangerous drivers reported being involved in more crashes within the past 3 years than the safe drivers did. Moreover, there were also significant differences between the two groups in their DDDI total scores (*t*(36) = 3.906, *p*<0.001) and in the scores for all DDDI subscales. General sociodemographic information for all 38 participants is presented in Table 1.

**Table 1:** *DDDI* and sociodemographic information for the two groups of drivers.

	Dangerous (n = 15)	Safe (n = 23)	*t*/χ^2^
*Demographic variables*			
Age (years)	30.47 (4.76)	33.61 (6.72)	*t* = 1.569
Education (years)	15.80 (1.90)	15.17 (2.31)	*t* = 0.874
Gender (% male)	46.67%	52.17%	χ^2^ = 0.110
*Driving experience*			
Driving years	5.57 (2.56)	6.96 (4.11)	*t* = 1.169
Total mileage (10,000 km)	6.63 (4.34)	7.34 (5.83)	*t* = 0.404
Weekly mileage (km)	268.47 (302.07)	360.00 (510.45)	*t* = 0.625
*Driving violation history*			
Penalized points for			
- Speeding	4.00 (3.34)	0.13 (0.63)	*t* = 4.439**
- Running a red light	1.20 (1.90)	0.00 (0.00)	*t* = 2.449**
Number of Crashes	4.33 (2.38)	1.43 (1.90)	*t* = 4.156**
*Dangerous driving behavior*			
DDDI total scores	85.73 (24.81)	59.04 (11.40)	*t* = 3.906**
-RD	34.13 (7.36)	22.87 (5.54)	*t* = 5.381**
-AD	15.73 (4.10)	12.04 (2.82)	*t* = 3.295*
-NCED	27.33 (4.75)	21.35 (3.90)	*t* = 4.243**
-DD	4.20 (1.82)	2.30 (0.64)	*t* = 3.882**

Note:

* *p*<0.05

** *p*<0.01

### 3.2 Behavioral data from the emotional Stroop task

**Fig 1:**
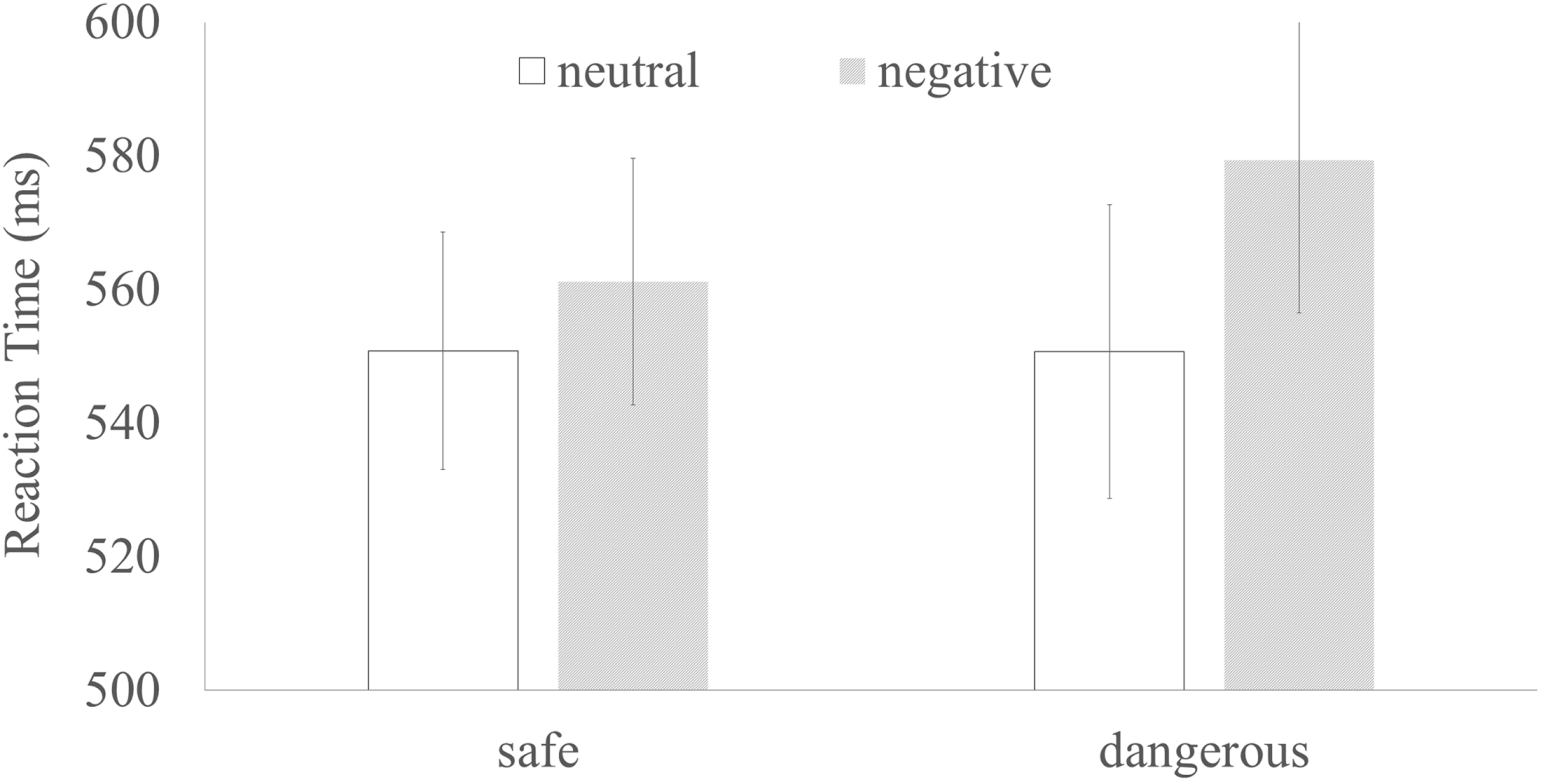
The mean reaction times (ms) for the emotional Stroop task under each condition. Error bars are included.

Mixed-design ANOVAs of emotion (negative vs. neutral) by group (dangerous drivers vs. safe drivers) were applied to the participants’ reaction times and accuracy in the emotional Stroop task. Both groups’ mean reaction times and accuracy in the emotional Stroop task are shown in Fig 1.

The reaction times in the emotional Stroop task showed a significant main effect of emotional pictures [*F*(1,36) = 19.097, *p*<0.001], indicating a strong and valid emotional bias among the participants. The reaction times of the dangerous drivers did not significantly differ from those of the safe drivers [*F*(1,36) = 0.101, *p* = 0.752]. However, the interaction between different emotional pictures and groups was significant [*F*(1,36) = 4.201, *p*<0.05]. Pairwise comparisons (Bonferroni) revealed that the reaction times for the negative pictures (M = 579.40, SD = 84.44) were longer than those for the neutral pictures (M = 550.72, SD = 73.02) in the dangerous driver group (*p*<0.001), but this pattern was statistically absent (negative: M = 561.19, SD = 91.09; neutral: M = 550.82, SD = 91.89) among the safe drivers (*p* = 0.073); thus, the dangerous drivers exhibited greater emotional bias than the safe drivers did. The results revealed no main or interaction effects on accuracy between the dangerous drivers (negative: M = 0.97, SD = 0.03; neutral: M = 0.97, SD = 0.03) and the safe drivers (negative: M = 0.97, SD = 0.02; neutral: M = 0.98, SD = 0.03). An independent samples T test to compare the increases in reaction times between the dangerous drivers (M = 28.68, SD = 29.92) and the safe drivers (M = 10.36, SD = 24.82) also showed a significant difference between the groups (*t* = -2.050, *p* = 0.048).

### 3.3 Electrophysiological data during the emotional Stroop task

**Fig 2:**
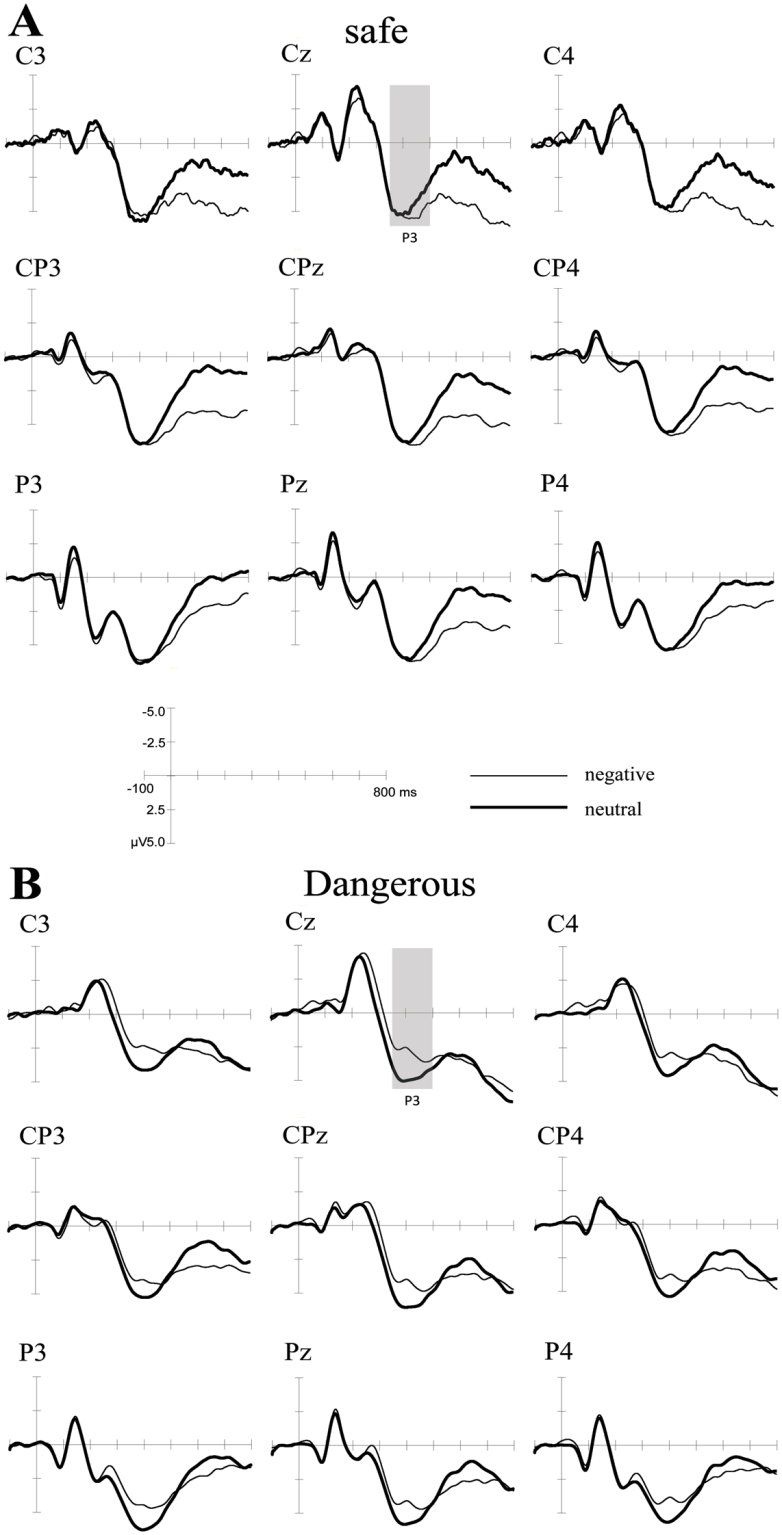
The grand-average ERPs for the different conditions. The figure depicts the ERPs for (A) the safe driver group and (B) the dangerous driver group. The time windows analyzed for the two effects in the four conditions are marked as grey boxes.

The mixed-model ANOVA revealed a significant interaction effect [*F*(1,36) = 5.873, *p*<0.05] on P3 amplitude. Pairwise comparisons revealed that only the dangerous drivers exhibited a significant difference between the two types of emotional stimuli (*p*<0.05). For the dangerous drivers, the mean amplitude of the response to negative stimuli (M = 3.84, SD = 0.80) was significantly smaller than the mean amplitude of the response to neutral stimuli (M = 4.93, SD = 0.73). No significant difference was observed for the safe drivers, and no significant group effect was found. The grand-average ERPs for the different conditions were shown in Fig 2.

### 3.4 Additional analyses

The negativity bias score, which is often calculated as the mean reaction time for the negative stimuli minus that for the neutral stimuli (e.g., Putman et al, 2004; Honk et al, 2001), is a commonly used index of the interference caused by emotional stimuli. In this section, we used the negativity bias score as an important variable to study the relationships between negativity bias and driving behaviors.

First, a Pearson correlation analysis (two-tailed) revealed a marginal correlation (*r* = -0.310, *p* = 0.058) between the negativity bias scores (mean reaction time for the negative stimuli minus that for the neutral stimuli) and the P3 amplitude on Cz (i.e., the negative minus neutral stimuli). The correlation between the negativity bias score and self-reported driving behavior was tested. The results revealed that the emotional bias score was significantly correlated with the score on the NCED subscale of the DDDI (*r* = 0.459, *p*<0.01) and crash numbers in the last three years (*r* = 0.392, *p*<0.05).

In order to test the relationship among negative bias, dangerous driving behavior and crashes, we did a mediating effect analysis followed the four steps outlined by Baron and Kenny [69]: Step 1, test the effect of negative bias on crashes to show that an effect exists that may be mediated; Step 2, test the relationships between negative bias and dangerous driving behavior to show that negative bias is correlated with the mediator; Step 3, test the effect of dangerous driving behavior on crashes while controlling for demographic variables and negative bias to show that the mediators (negative driving behavior) influence crash involvement; and Step 4, test the effect of negative bias on crashes while controlling for dangerous driving behavior—if the effect disappears, the relationship between negative bias and crashes is completely mediated by the examined dangerous driving behaviors.

The main results of the mediating effect analysis are shown in Table 2. Negative bias significantly predicted crash numbers (Model 1), satisfying step 1. Then, in the regression of negative bias on NCED, negative bias was found to be a significant predictor of NCED after controlling for demographic variables (β = 0.465, p < 0.01), satisfying step 2. In step 3, crash numbers was regressed on negative bias and NCED in the model (Model 2). The relationships between NCED and crash numbers was significant, satisfying step 3. Moreover, the effect of negative bias on crash numbers was no longer significant, satisfying step 4. The results showed the effect of negative bias on crash involvement was totally mediated by NCED.

**Table 2:** Hierarchical multiple regression models' standardized regression coefficients (*β)*.

	Model 1	Model 2
	Beta	Beta
Negative bias	0.347**	0.099
NCED		0.533**
ΔR^2^	0.108**	0.214**
Model adjusted R^2^	0.078	0.297

Note: All regressions were adjusted for age, gender, and number of years driving. Model 1: negative bias as a predictor of crash numbers. Model 2: negative bias and NCED as predictors of crash numbers. *β* values were derived from the final step of each model.

** p < .01.

**Fig 3:**
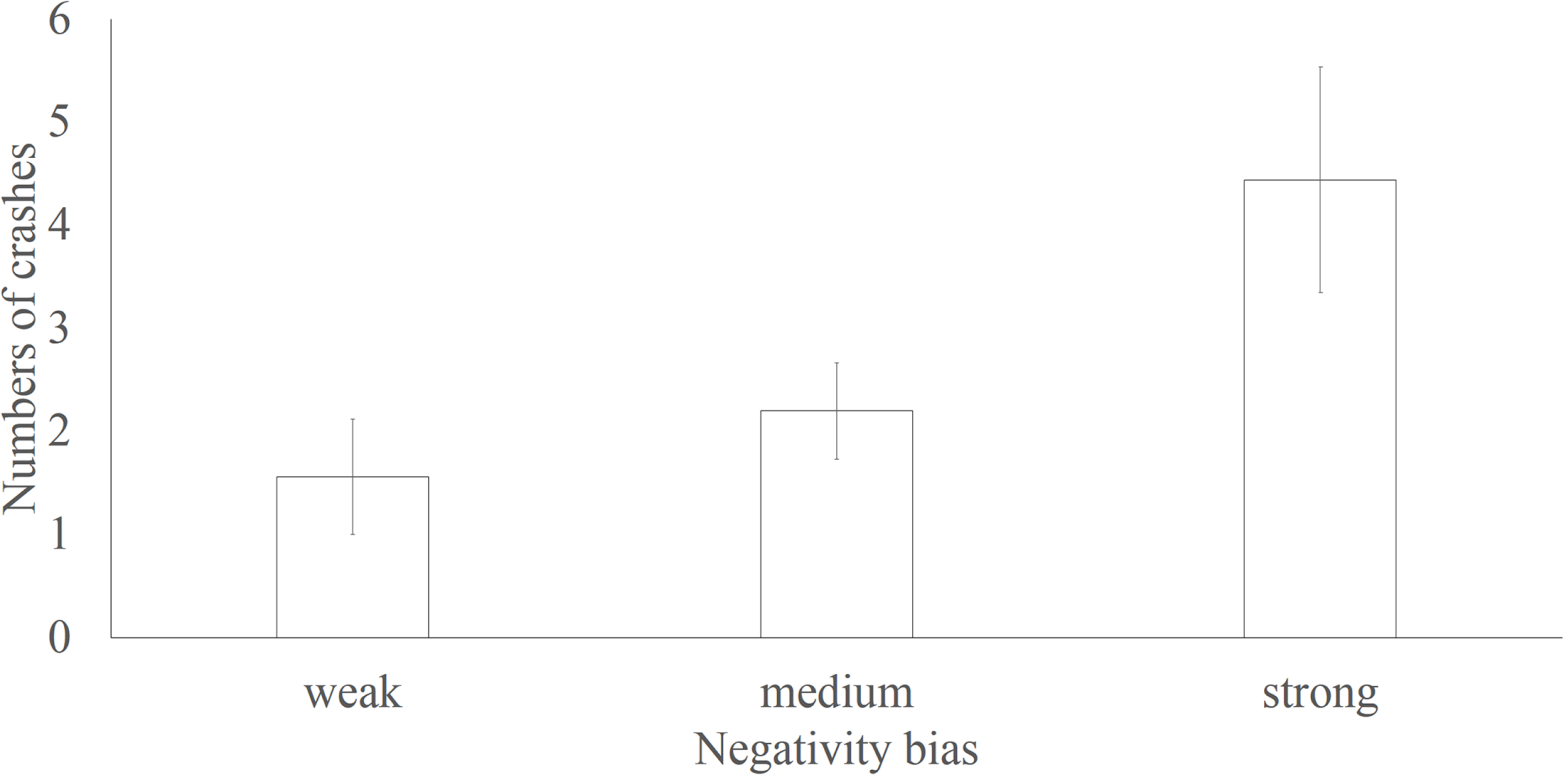
The number of previous crashes in each of the three groups and negativity bias. Error bars are included.

We divided the participants into three groups according to their negativity bias scores: the participants with the highest 25% (exceeding 38.31 ms) of scores were placed in the strong bias group, those with the lowest 27% (less than 1.14 ms) were placed in the weak bias group, and the remaining participants were placed in the medium bias group. A one-way ANOVA of these three groups revealed a significant difference in the number of crashes [*F*(2,35) = 3.983, *p*<0.05]. The results are shown in Fig 3. Multiple comparison tests (Bonferroni) revealed a significant difference (*p*<0.05) in the number of crashes between the strong bias group (n = 10, M = 4.44, SD = 3.28) and the weak bias group (n = 10, M = 1.56, SD = 1.67); no significant difference was found between the medium bias (n = 18, M = 2.20, SD = 2.09) and weak bias groups or between the medium bias group and the strong bias group. According to participants’ self-report, the crashes were divided into whether the participant was a victim or being at fault. The results showed that the numbers of crashes caused by the participants was significantly correlated with the negativity bias scores (*r* = 0.401, p = 0.013), however, no correlation was found between the numbers of crashes lead by others and the negativity scores (*r* = 0.120, p = 0.473).

## Discussion

This study examined the emotional information processing of dangerous drivers and safe drivers. The results revealed that dangerous drivers exhibited stronger negativity biases than safe drivers did. The P3 for negative stimuli was smaller than that for neutral stimuli in the dangerous driver group. Additionally, the emotional bias score was correlated with the frequency of negative emotional driving behaviors, as measured with the NCED subscale of the DDDI. Moreover, the drivers who exhibited stronger negativity biases also reported being involved in more crashes within the past three years than did the drivers with lower negativity biases. These results suggest a significant difference in the negativity biases of dangerous drivers and safe drivers.

The behavioral results indicated that dangerous drivers had stronger negativity biases than the safe drivers did based on their longer reaction times to the negative stimuli than to the neutral stimuli. This result indicates that dangerous drivers were more sensitive to negative information. A previous study found that individuals with a higher negativity bias were more likely to evaluate a situation as worse than it actually was and thus to overreact [37]. Another study found heightened sensitivity to anger-related stimuli causes drivers with anger-related traits to overreact to low-anger-provoking situations, which leads to increased speeding and overtaking behaviors [34]. Drivers with stronger anxiety-related traits are more likely to be involved in traffic tragedies than other drivers are, as stronger negativity biases may lead to overreaction [70–71]. These results could help us to explain why dangerous driver revealed negative bias.

The ERP results provided additional explanations for the negative information processing mechanisms of dangerous and safer drivers. The amplitudes of P3s elicited by negative stimuli were significantly smaller than those elicited by neutral stimuli only for the dangerous drivers; the safe drivers exhibited no significant difference. This contrast revealed a difference in how the two groups process emotional information. Some studies have also found reduced P3s for negative stimuli [47, 52, 56]. Reduced P3s might be indicative of a decrease in the inhibitory processing involved in controlling features that are irrelevant to the task but emotionally salient [47, 53, 58]. In our research, decreased inhibitory control was found only for the dangerous drivers, which might indicate that the dangerous drivers were more likely to be attracted to task-irrelevant information, which reduced their cognitive resources and thus impaired their task performance.

A significant positive correlation was found between the NCED subscale scores and the negativity bias scores, which provides valuable evidence of the relationship between negative stimuli processing and negative emotions during driving. The NCED subscale measures negative emotions experienced while driving, such as frustration, anger and rumination, which can distract individuals from devoting the proper attention to driving [1, 2]. Drivers with a higher negativity bias reported more negative emotions while driving. They may be more sensitive to angry or frustrating stimuli while driving, and the negativity bias might explain their response to negative input and behavior.

Interestingly, a significant relationship between negativity bias and the number of crashes within the past three years was also found. The strong bias group had been involved in a greater number of crashes than the weak bias group had. Previous studies have found that drivers with certain characteristics (e.g., higher trait anger) are more likely to be involved in crashes and to commit violations [17]. These drivers also tend to exhibit a stronger emotional bias [32, 33]. But the effect of negative bias on crashes was mediated by negative cognitions and emotions during driving. As the small sample of our research, future research could explore this issue deeply. However, previous research has not explored the relationship between emotional bias and driving outcomes (e.g., traffic crashes). Our results filled this gap in the literature by directly examining the relationship between previous crashes and negativity bias.

There are some limitations to this research. First, the dangerous drivers in our study were screened based on their penalty points and DDDI scores, the crash number were measured by self-reported data, the actual crashes and violations recorded by traffic management departments could provide more precise data. Insurance company records could also be useful. Second, the small sample of participant limited the generalizability of our conclusions to the entire population of drivers in China. Besides, using the penalty points as the cutoff index may have some limitation as the points were penalized by different reason. Future studies could exam drivers with same violation which could increase risk of injury and death from crashes, such as speeding and red light running. Additionally, the present study revealed only a correlation between negativity bias and crashes; the cause and effect between crashes and negativity bias were still unclear, and the relationship between negativity bias and crashes involvement requires further investigation. Future research could examine the effect of negativity bias on online crashes, such as in driving simulations.

## Conclusion

In conclusion, we observed stronger negativity biases in dangerous drivers than in safe drivers. The bias score was correlated with dangerous driving behaviors. The drivers with a strong negativity bias reported more extensive crash histories compared with the drivers with lower levels of bias. The ERP results showed that dangerous drivers exhibited reduced P3s in response to negative stimuli, which revealed a decreased inhibitory control of information that is task irrelevant but emotionally salient. The influence of negativity bias provides one possible explanation for the effects of individual differences on dangerous driving behavior and traffic crashes.

## Supporting Information

S1 File Behavior data for negativity bias. (XLSX)
